# Supplementary material for: Genetic comparisons of interleukin-17 reveal a framework for complex signaling evolution
Source: bioRxiv. 2026 Apr 14:2026.04.13.718218. Preprint. [Version 1] doi: 10.64898/2026.04.13.718218 (PMC13105070; doi:10.64898/2026.04.13.718218)
Supplement: Supplement 3 [file media-3.zip › Supplemental files for paper/ERC/IL17xIL17_comparisons/IL17RD_IL17F.pdf]

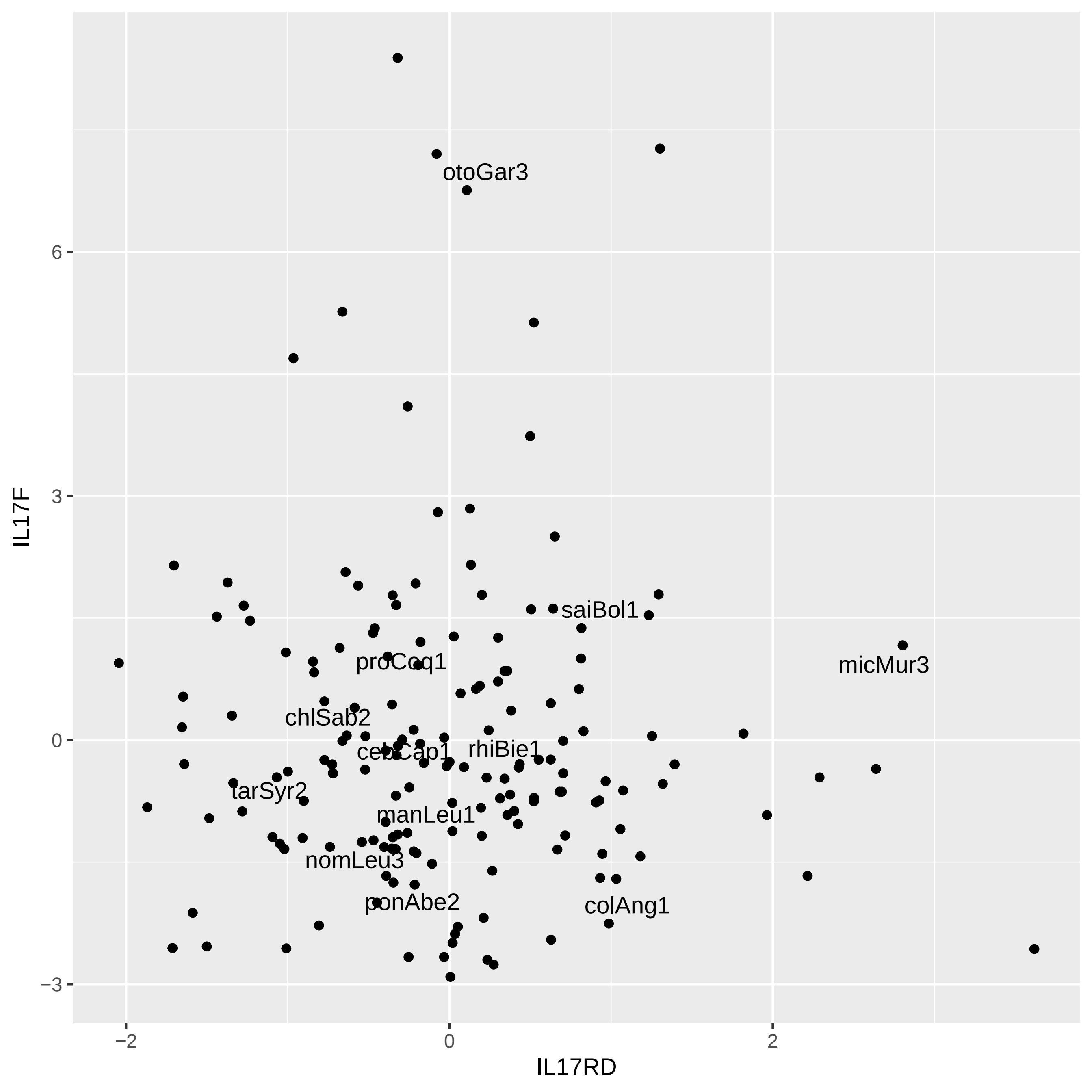

otoGar3
saiBol1
micMur3
proCoq1
chlSab2
cebCap1
rhiBie1
tarSyr2
manLeu1
nomLeu3
ponAbe2
colAng1
IL17F
IL17RD
6
3
0
−3
−2
0
2
